# Electrocardiogram lead selection for intelligent screening of patients with systolic heart failure

**DOI:** 10.1038/s41598-021-81374-6

**Published:** 2021-01-21

**Authors:** Yu-An Chiou, Jhen-Yang Syu, Sz-Ying Wu, Lian-Yu Lin, Li Tzu Yi, Ting-Tse Lin, Shien-Fong Lin

**Affiliations:** 1 Department of Electrical and Computer Engineering, College of Electrical and Computer Engineering, National Chiao-Tung University, Hsinchu, Taiwan; 2 Institute of Biomedical Engineering, College of Electrical and Computer Engineering, National Chiao-Tung University, 1001 University Road, Hsinchu, Taiwan; 3 Division of Cardiology, Department of Internal Medicine, National Taiwan University BioMedical Park Hospital, Hsinchu City, Hsin-Chu County Taiwan; 4 Department of Internal Medicine, College of Medicine, National Taiwan University, Taipei, Taiwan; 5 Department of Nursing, National Taiwan University Hospital Hsinchu Branch, Hsin-Chu, Taiwan

**Keywords:** Heart failure, Machine learning

## Abstract

Electrocardiogram (ECG)-based intelligent screening for systolic heart failure (HF) is an emerging method that could become a low-cost and rapid screening tool for early diagnosis of the disease before the comprehensive echocardiographic procedure. We collected 12-lead ECG signals from 900 systolic HF patients (ejection fraction, EF < 50%) and 900 individuals with normal EF in the absence of HF symptoms. The 12-lead ECG signals were converted by continuous wavelet transform (CWT) to 2D spectra and classified using a 2D convolutional neural network (CNN). The 2D CWT spectra of 12-lead ECG signals were trained separately in 12 identical 2D-CNN models. The 12-lead classification results of the 2D-CNN model revealed that Lead V6 had the highest accuracy (0.93), sensitivity (0.97), specificity (0.89), and f1 scores (0.94) in the testing dataset. We designed four comprehensive scoring methods to integrate the 12-lead classification results into a key diagnostic index. The highest quality result among these four methods was obtained when Leads V5 and V6 of the 12-lead ECG signals were combined. Our new 12-lead ECG signal–based intelligent screening method using straightforward combination of ECG leads provides a fast and accurate approach for pre-screening for systolic HF.

## Introduction

Heart failure (HF) is a prevalent cardiovascular condition and a considerable public health problem^1,2^. Half of the patients with HF have systolic cardiac contractile dysfunction, which is usually confirmed by ejection fraction (EF) measured with echocardiography^3^. Although the echocardiographic diagnosis of systolic HF is precise, this modality is relatively time-consuming and costly in comparison with electrocardiography^4^. By contrast, a 12-lead electrocardiogram (12-lead ECG) is a convenient and inexpensive tool that provides comprehensive information on cardiac electrical dynamics. Consequently, its use is highly desirable for the early screening of suspected systolic HF.

In recent years, artificial intelligence (AI) has been widely used in the medical field^5,6^. Numerous AI ECG-based feature detection approaches have been employed, such as the use of artificial neural networks^4^, image classification–based convolutional neural networks (CNNs)^7^, time-relative recurrent neural networks^8^, and the unsupervised method^9^. Along with the rapid development of AI, studies on using ECG signals to prescreen for specific diseases based on AI algorithms are evolving rapidly. Crucial research on systolic HF combine 12-lead ECG for classification was markedly progressed. However, a single ECG lead or combination of leads primarily drove accuracy of EF discrimination is unknown. In this study, we applied the continuous wavelet transform (CWT) to convert the 1D-ECG signals to 2D spectra for 2D-CNN classification. The contribution of individual ECG leads to the classification result was evaluated, and a comprehensive scoring method was designed to improve outcomes.

## Methods

### Dataset

This study was approved by the Research Ethics Committee for Human Subject Protection, National Taiwan University Hospital, Hsinchu Branch, Hsinchu, Taiwan (IRB number:108-073-E), and each person enrolled were all over 18 years old and gave written informed consent to participate. All authors confirm that all the experiments were performed in accordance with relevant guidelines and regulations. Two datasets were used in this study. One included 1090 systolic HF patients with an EF of < 50%. The other included 10,000 individuals with an EF of > 50% and without HF symptoms. The EF was measured by echocardiography performed by cardiologists, and 12-lead ECG data were acquired at clinics or during hospitalization. Both datasets were provided by National Taiwan University Hospital, Hsinchu and Biomedical Park Branch. The 12-lead ECG data of all participants were obtained within one week after echocardiography identified their left ventricular EF (LVEF) greater than 50% or not. Each 12-lead ECG recording was from a single participant, without duplication.

### Patient selection

**Figure 1:**
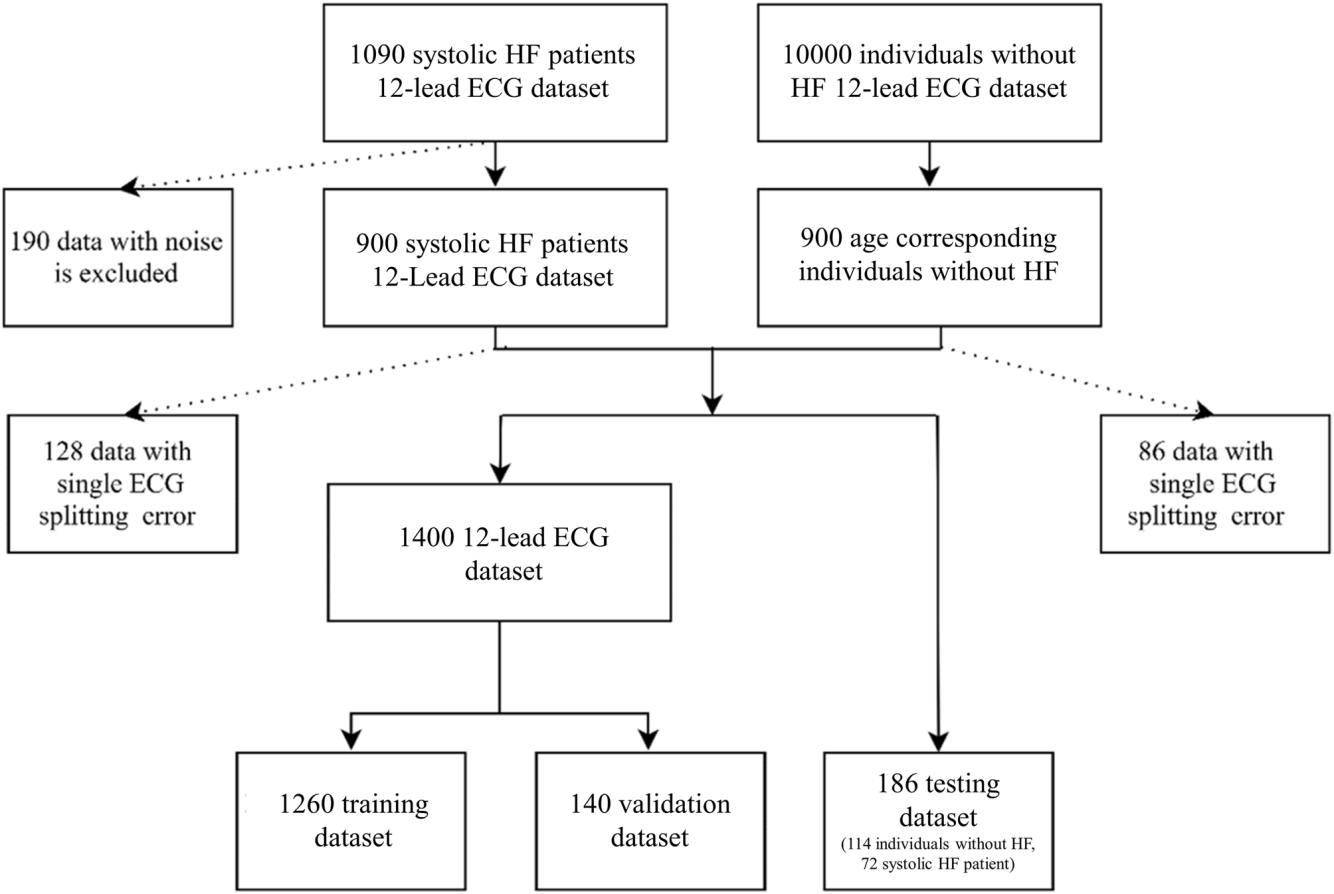
Data selection flowchart. A total of 900 patients with systolic HF were included in the study. For comparison, 900 age-matched individuals without systolic HF were included in the research. After ECG preprocessing, noisy data and data with ECG splitting errors were excluded. The remaining 700 patients with systolic HF and the corresponding individuals without HF were combined into one dataset. These data were then separated into groups of 1260 for training data, 140 for validation data, and 186 for testing data.

**Figure 2:**
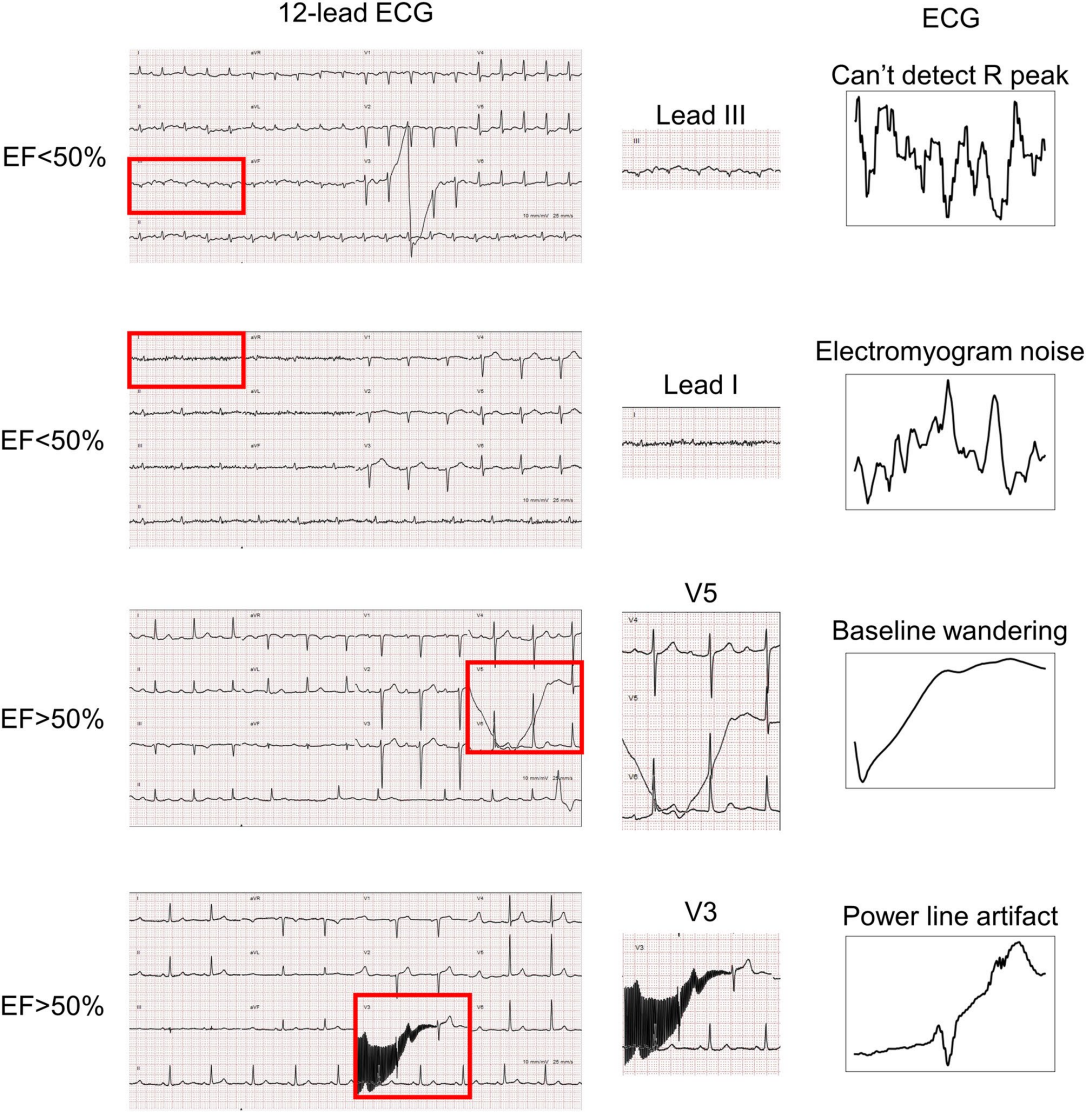
Signals with excessive ECG noise, such as R peak detect error, electromyogram noise, baseline wandering and power line artifact, were excluded from our study. These ECG signals are severely affected by noise and therefore cannot be processed to obtain the correct ECG compound. They were excluded from our research to avoid misleading the neural network model during training.

The patient selection process is presented in Fig. 1. Among the 1090 patients with reduced EF, 12-lead ECG data with excessive noise were excluded from this research. ECG data with excessive noise was attributed to interferences from baseline wander, power line interference, electromyography noise, and R peak detection error. Examples of noise illustrations were shown in Fig. 2. Excessive noise in the ECG signal resulted in a splitting error. Splitting errors may generate an atypical waveform map, which could mislead our model for finding EF features. Thus, such ECG signals were excluded from our study. The remaining 12-lead ECG data for 900 patients with systolic HF was used as the patient training dataset. The corresponding 900 age-matched and EF-normal individuals were selected from the dataset with 10,000 individuals from health examination. Information for the patients with systolic HF and the individuals without HF are presented in Table 1. A total of 214 individuals were excluded due to having ECG data with signal splitting errors. A total of 186 testing data were randomly selected from the remaining 772 patients with systolic HF and 814 individuals without HF. The data of the systolic HF patients and the individuals without HF (total 1400) were randomly separated into two groups: 90% of data were used for training (n = 1260) and 10% of data were used for validation (n = 140). All patient’s original EF values were measured with echocardiograph. The systolic HF patient (EF < 50%) and individuals without systolic HF (EF > 50%) were divided in two classes, and compared to the AI prediction class.

**Table 1:** Information on individuals with and without systolic HF.

	Systolic HF patients	Individuals without HF
Number	900	900
Male	630	417
Female	270	483
Age (years)	69.3 ± 14.6	71.4 ± 13.7
Height (cm)	162.1 ± 9.7	158.9 ± 10.3
Weight (kg)	61 (76–51)	66 (52–83)
LVEF (%)	34 (24–42)	61 (54–76)
LVEDD (mm)	53 (46–61)	43 (39–51)
LVESD (mm)	46 (42–56)	31 (24–40)
iVS (mm)	9 (6–13)	8 (6–12)
PWD (mm)	11 (8–14)	9 (7–12)
E/A (ratio)	0.7 ± 0.5	1.1 ± 0.4

### Electrocardiogram extraction

**Figure 3:**
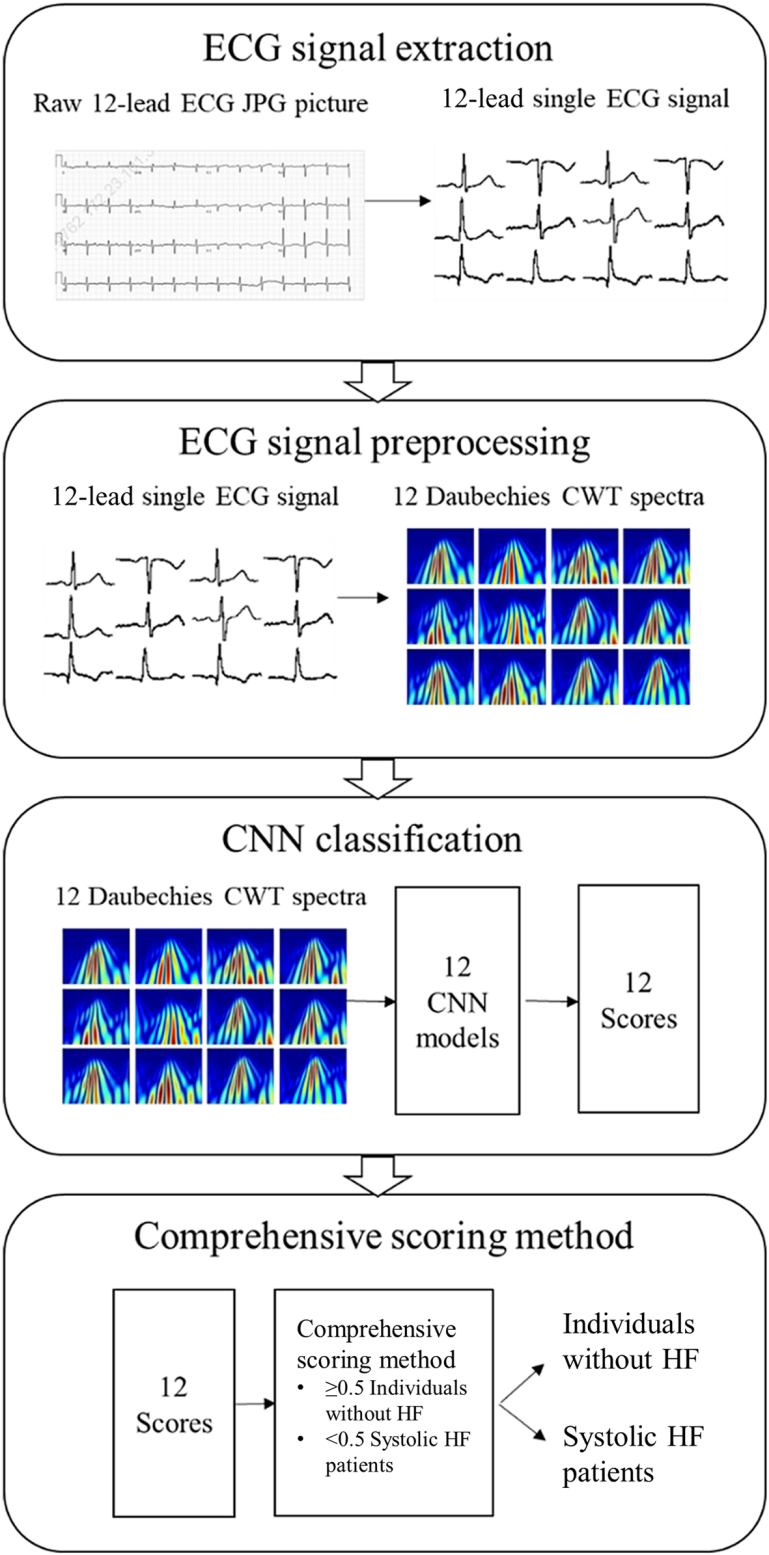
Illustration of the research process employed in this study. The first step was to extract the ECG signal from the JPG images. The second step was to transform each single-lead ECG signal into CWT spectra. In the final step, the spectra were trained separately in 12 models for the 12-leads, and the softmax layer output scores (ranging from 0 to 1) were recorded and applied for the comprehensive scoring method. Four comprehensive scoring methods were considered, including one where equal weights were given to the 12-leads and the key leads close to the left ventricle (Leads I, V5, and V6).

**Figure 4:**
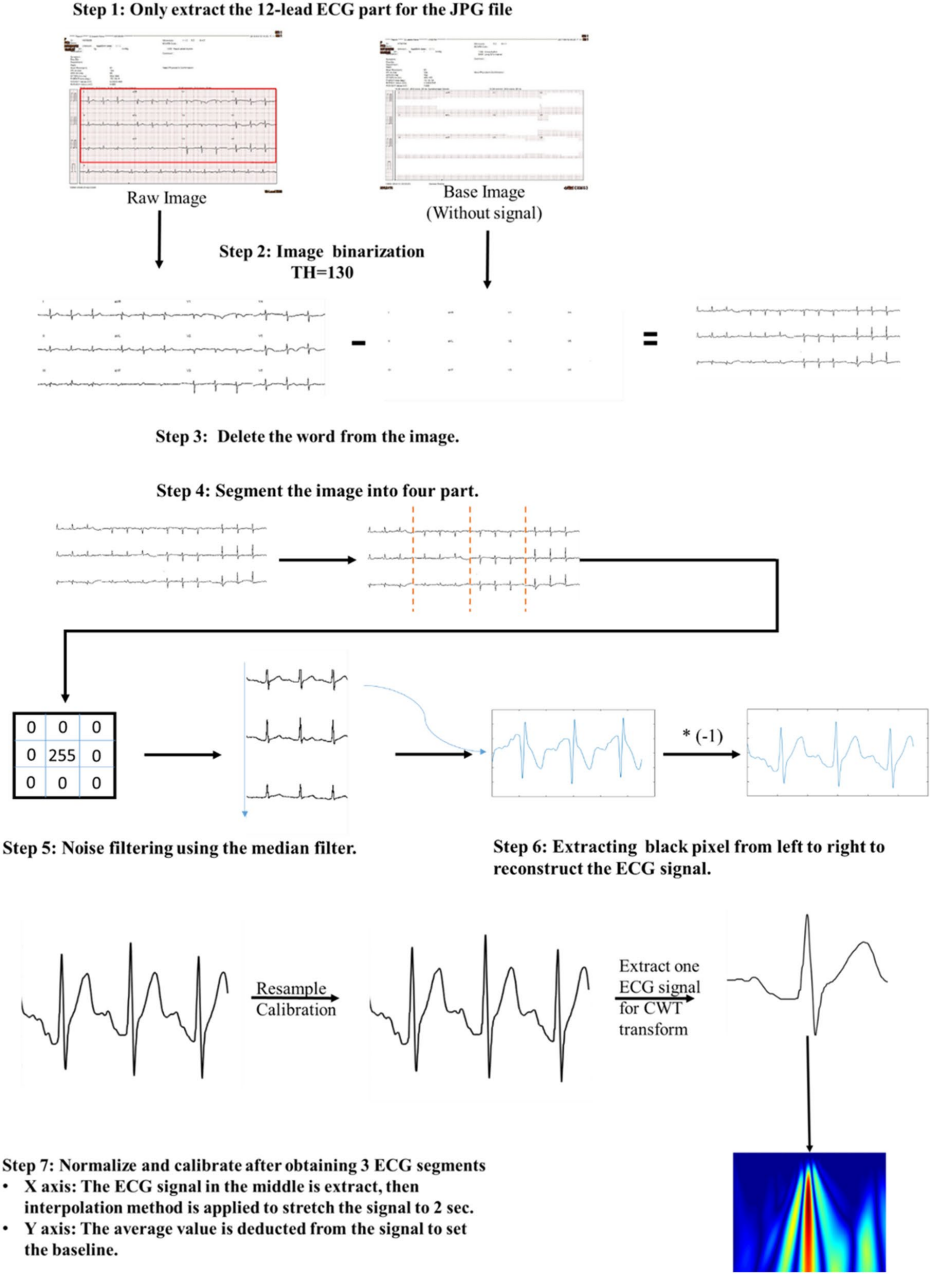
12-lead ECG signal preprocessing. Details are provided in seven steps. Before converting the 12-lead ECG images into the signal, it was necessary to confirm the target area of the ECG signal and then crop the region containing the ECG image and transform the RGB image into a grayscale image. Image binarization could convert the color of the signal and background into black or white. Then, the pixel bits of each lead signal were regarded as coordinates: X, regarded as time, and Y, regarded as relative size, were independently accessed. The squares on the 12-lead ECG images were used as the basis for determining the length of time required to reconstruct the original signal. Finally, the interpolated signal was used to compare the relative position of the pixel to the time required to reconstruct the electrical signal and the signal’s size.

The flow chart for the whole experiment, including ECG extraction, CWT, and 2D-CNN classification, is depicted in Fig. 3. Because the 12-lead ECG data were recorded as a JPG image, the ECG signals had to be extracted from the image. The extraction procedure involved processing the JPG image through image binarization and signal extraction to obtain pure ECG signals. Then, the ECG image was cut vertically into four parts, followed by searching for black pixels on each of three vertical line to reconstruct the original ECG signal. The reconstructed ECG signal was then normalized and calibrated. Each ECG line was cut between two R peaks to obtain three small segments, and the middle segment was selected as the single-beat ECG compound. This procedure generated 12 single-lead ECG compounds for further processing. The details of the ECG extraction process are demonstrated in Fig. 4.

### Continuous wavelet transform

The 12-lead ECG signals were transformed by CWT to 2D spectra. Wavelet transform can be used to analyze time series in different frequencies that contain nonstationary power. In this research, Daubechies CWT (db8) was used to transform the ECG signals because it has a favorable balance between time and frequency localization. The CWT and Daubechies wavelet formulas are shown in Eqs. (1) and (2).

CWT equation:

1 $$ {\text{CWT}}_{{\text{x}}}^{\uppsi } \left( {\tau ,s} \right) = \frac{1}{\sqrt s }\int x \left( t \right)\uppsi ^{*} \left( {\frac{t - \tau }{s}} \right){\text{d}}t $$

Daubechies equation:

2 $$ {\text{H}}_{\emptyset } \left( {{\text{e}}^{{{\text{j}}\upomega }} } \right) = \sqrt 2 \left( {\frac{{1 + {\text{e}}^{{ - j\upomega }} }}{2}} \right)^{p} R\left( {{\text{e}}^{{{\text{j}}\upomega }} } \right) $$

In the CWT Eq. (1), τand s correspond to the translation and scale parameters, respectively. ψ(t) is the transforming function, which also represents the mother wavelet. Matlab CWT toolbox was employed in the CWTs. In the Daubechies Eq. (2), $p$ represents a vanishing moment^10^. The Matlab wavelet toolbox was used for the implementation of these two equations.

### CNN structure

The neural network programming was based on the Python and Keras application programming interfaces. The 2D-CNN structure was modified from the Visual Geometry Group (VGG) network^5^ for the 12-lead ECG CWT spectra classification. The 12-lead ECG CWT spectra were first resized to 200 × 200 × 3 pixels and then passed to the 2D-CNN as inputs. A 14-layer 2D-CNN was constructed with 6 convolution layers, 6 max-pooling layers, 1 flatten layer and 1 dense output layer with softmax function. The rectified linear unit, batch normalization, and dropout functions were used after each convolution layer was applied. Binary cross-entropy was defined as the loss function. An Adam optimizer was employed as the learning guide for 2D-CNN learning, and its learning rate was set as 10^–4^. For detailed 2D-CNN structure and hyperparameter information, please refer to Tables S1 and S2. In this research, the 12-lead ECG spectra were separately passed to 12 identical 2D-CNNs.

### Comprehensive 12-lead ECG scoring

The comprehensive 12-lead ECG scoring method is based on the 2D-CNN output layers with a softmax formula, which is displayed in Eq. (3).

3 $$ {\text{S}}\left( {{\text{yi}}} \right) = \frac{{{\text{e}}^{{{\text{yi}}}} }}{{\sum\limits_{j} {e^{{y_{j} }} } }}\quad for\;i \, = \, 1, \ldots j $$

The logits vector from the 2D-CNN flatten layer proceeds through the softmax layer, the output class probability score of i(yi), and the class probability score summation [$\sum\nolimits_{j} {e^{{y_{j} }} }$ (for j from 0 to 1)]^6^. In our research, the 12-lead ECG CWT spectra were separately passed to 12 2D-CNNs, which generated 12 probability scores in the individual without HF class. The 12 probability scores were employed in our comprehensive 12-lead ECG scoring method.

This method integrates 12 scores into one key diagnostic index for detecting systolic HF. V5, and V6 are the three ECG leads physically closest to the left ventricle, and they may have more relevance to EF detection than other ECG leads. Also, the Leads I result shows higher accuracy (82%) than Lead II and Lead III, so this lead had been considered in our comprehensive method. The scores from these three leads were selected in our comprehensive scoring method. The four types of scoring method were designed to obtain four diagnostic indices. The first one is the average value of the 12-lead output score, named “12-lead with equal weighting;” the second index is the average of three crucial lead scores, Leads I, V5, and V6, named “Lead I, V5, and V6”; the third index is the average value of Lead I and V6 scores, named “Lead I and V6;” and the fourth index is the average value of the V5 and V6 scores, named “V5 and V6.” The 12 output prediction scores from 12 neural network softmax layers were summed and used the cutoff value of 0.5. If the summation score is greater than or equal to 0.5, the individual EF is in the normal range (≧ 50%). By contrast, a summation score below 0.5 indicates that the individual has low EF (< 50%).

### Statistical method

Descriptive continuous data were presented as mean ± standard deviation if normal distributed or otherwise as median/IQR. Ejection fraction measured by echocardiography were compared with comprehensive scoring and 12-lead 2D-CNN scoring predictions using accuracy, sensitivity, specificity, and f1 score formulas. These formulas can be used to evaluate the predictive capability of the 12-lead ECG, 2D-CNN model as well as the true positive (TP), true negative (TN), false positive (FP), and false negative (FN) rates^11^. These formulas are written as follows: accuracy = (TP + TN)/(TP + TN + FP + FN), sensitivity = TP/ (TP + FN), specificity = TP/(TP + FN), precision = TP/(TN + FP), recall = TP/(TP + FN) and f1 score = (2 × precision × recall)/(precision + recall).

The receiver operating characteristic curve (ROC) was used as an evaluation method in this study. The ROC curve is a common analysis method for evaluating deep learning models. Using ROC curves, the graphical display of true positives (as the y-axis) versus false positives (as the x-axis) can be observed and compared directly. The area under the ROC curve (AUC) represents the equivalent of the probability when randomly selecting a sample. The classifier ranks a randomly chosen positive sample higher than a randomly chosen negative sample. The AUC value ranges between 0 and 1, and if the AUC value is in the range of 0.5 < AUC < 1, it means the classifier has more effective predicting ability than random guesses^12^.

## Results

### Systolic HF prediction results

**Figure 5:**
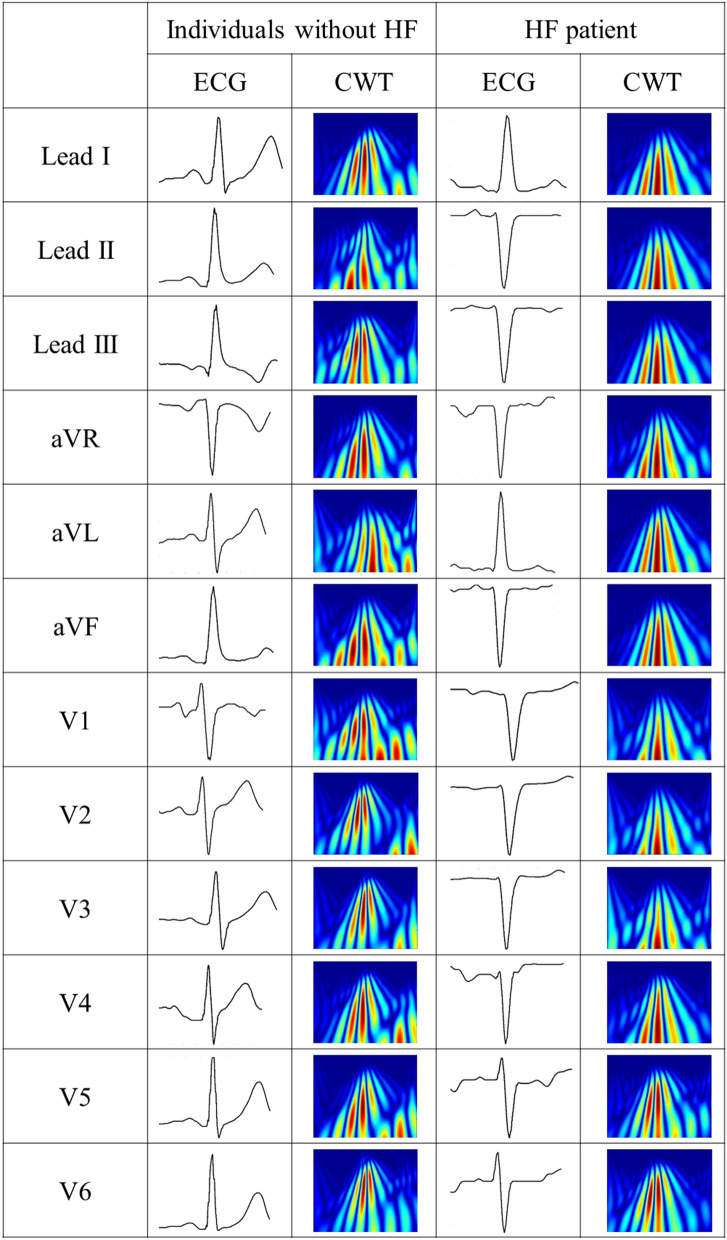
Illustration for the CWT of the 12-lead ECG. The original image of the ECG JPG file and CWT spectra were showed.

The 12-lead ECG data of the 900 HF patients and the 900 individuals without HF were transformed into CWT spectra. The baseline data of individuals with and without systolic HF are listed in Table 1. Furthermore, Table 2 presents the characteristics of the training, validation, and testing sets. In the testing dataset (n = 186), the mean LVEF was 32.6 ± 3.4%, and 73 (39.2%) patients had myocardial infarction, 82 (44.1%) had hypertension, and 69 (37.1) had diabetes. Those values were similar to those in the training dataset (LVEF = 32.3 ± 4.6, *p* = 0.25; myocardial infarction 36.7%, *p* = 0.16; hypertension 48.5%, *p* = 0.09, and diabetes 37.0%, *p* = 0.11). Figure 5 illustrates the original ECG data from the JPG image and the CWT spectra. The CWT spectra can concentrate the unobvious ECG linear features into 2D image, which can enhance specific features of HF for machine learning classification. Also, the tenfold cross validation had been applied to our model, and demonstrated that the V6 had the highest average accuracy of 89.07% (Table S3).

**Table 2:** Characteristics of the training, validation, and testing sets.

	Training set (n = 1260)	Validation set (n = 140)	Testing set (n = 186)	*P* value
Female, n (%)	629 (49.9)	59 (42.1)	65 (34.9)	–
Age, years	70.5 ± 10.9	69.7 ± 11.0	70.2 ± 11.1	0.46
Age group, n (%)	–	–	–	–
< 40	40 (3.2)	7 (5.0)	10 (5.4)	–
40–49	72 (5.7)	8 (5.7)	14 (7.5)	–
50–59	275 (21.8)	34 (24.2)	42 (22.6)	–
60–69	373 (29.6)	41 (29.3)	49 (26.4)	–
70+	500 (39.7)	50 (35.8)	71 (38.1)	–
Mean EF	32.3 ± 4.6	31.8 ± 4.8	32.6 ± 3.4	0.25
Diabetes	467 (37.0)	54 (38.5)	69 (37.1)	0.11
Hyperlipidemia	545 (43.2)	64 (45.7)	79 (42.4)	0.26
Hypertension	612 (48.5)	71 (50.7)	82 (44.1)	0.09
Renal disease	189 (15.0)	20 (14.3)	31 (16.6)	0.23
Myocardial infarction	462 (36.7)	51 (36.4)	73 (39.2)	0.16

### Systolic HF prediction results for individual leads

A total of 1400 ECG training data and 186 ECG testing data were used in this study. The accuracy, sensitivity, specificity, and f1 score of the test dataset are revealed in Table 3. The ECG results for individual ECG leads were favorable for the classification of patients with systolic HF. Each lead had accuracy ranging from 0.71 to 0.93. In particular, lead V6 exhibited the highest accuracy (0.93), specificity (0.97), and f1 score (0.94). The full results are presented in Table 3A.

**Table 3:** 2D-CNN classification results. (A) reveals the results of the 12-lead ECG scoring methods. (B) presents the results of four comprehensive 12-lead ECG scoring methods.

	Accuracy	Sensitivity	Specificity	F1score
**(A) 12-lead ECG each lead**				
aVF	0.80	0.78	0.82	0.81
aVR	0.77	0.82	0.72	0.80
aVL	0.84	0.96	0.70	0.87
Lead I	0.82	0.89	0.74	0.85
Lead II	0.80	0.83	0.77	0.82
Lead III	0.71	0.75	0.67	0.74
V1	0.77	0.79	0.76	0.79
V2	0.81	0.76	0.87	0.81
V3	0.76	0.93	0.56	0.81
V4	0.80	0.85	0.74	0.82
V5	0.81	0.88	0.73	0.84
V6	0.93	0.97	0.89	0.94
Average	0.80	0.85	0.75	0.83

	Accuracy	Sensitivity	Specificity	f1score
**(B) 12-lead ECG comprehensive scoring method**				
12-lead with equal weight	0.88	0.96	0.79	0.90
Lead I + V5 + V6	0.90	0.94	0.84	0.91
LeadI + V6	0.93	0.97	0.90	0.95
V5 + V6	0.94	0.97	0.89	0.94

### ROC analysis and comprehensive scoring results

**Figure 6:**
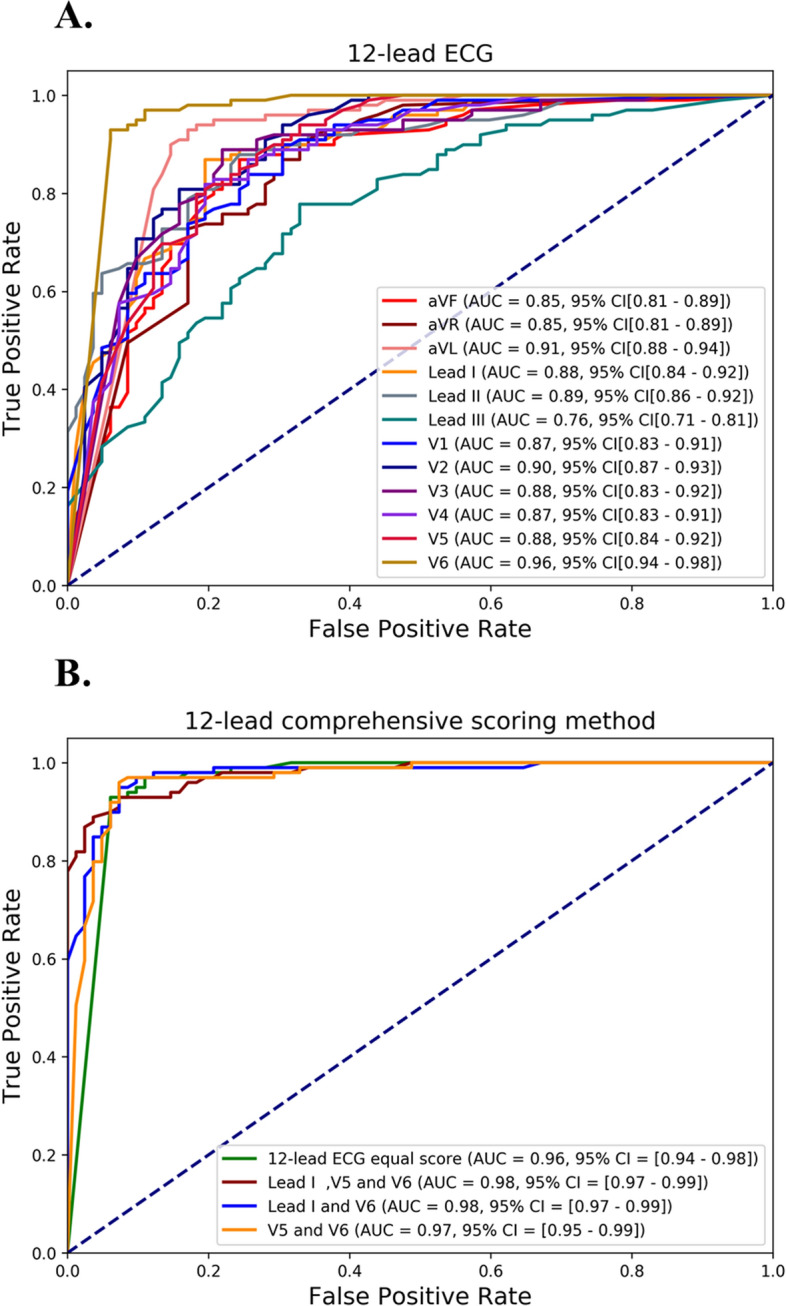
The ROC curves were presented. (**A**) presents a comparison of the 12-lead results for each lead and (**B**) presents the comprehensive scoring method results.

The ROC curve of our 12-lead ECG (with individual lead results) and comprehensive scoring results are detailed in Fig. 6. In Fig. 6A, the individual lead 2D-CNN models reveal AUC values between 0.76 and 0.96. Figure 6B reveals that the four AUC values for comprehensive scoring are between 0.96 and 0.98; these are higher than for any individual leads. The purpose of the comprehensive 12-lead ECG scoring method is to obtain one precise diagnostic index for systolic HF classification from 12 CNN models. Thus, the four comprehensive scoring methods were designed. The comprehensive scoring method’s accuracy, sensitivity, specificity, and f1 score are illustrated in Table 3B. The four comprehensive scoring method results include the “12-leads with equal weighting,” “Lead I, V5, and V6,” “Lead I and V6,” and “Lead V5 and V6.” In Table 3B, Lead V5 and V6 reveals the highest accuracy of 0.94, sensitivity of 0.97, specificity of 0.89, and an f1 score of 0.94. The four comprehensive scoring methods all showed higher accuracy, sensitivity, specificity, and f1 score than the average 12-lead ECG results.

## Discussion

A pre-screening systolic HF was established in this study. However, prospective testing of this method is still needed. The novel and comprehensive 12-lead ECG scoring method can achieve higher classification performance than individual leads. The individual leads had an average accuracy, sensitivity, and specificity of 0.75 and an f1 score of 0.83. However, the accuracy (0.94), specificity (0.97), sensitivity (0.89), and f1 score (0.94) all improved when the comprehensive 12-lead ECG scoring method was used.

In the individual lead results, V6 had higher accuracy than the other 11 leads. The screening results revealed that V6 was the representative lead of the 12-leads for pre-screening patients for systolic HF. This might be because V6 is the lead physically closest to the left ventricle. In comprehensive scoring, the four methods all had high classification capability. Among them, V5 and V6 had better classification capability than the other comprehensive scoring methods. In the ROC curve, the comprehensive scoring method exhibited significantly improved AUC compared with the AUC of the 12 individual leads. Therefore, V6 single lead and the comprehensive scoring method can be highly effective for screening patients for systolic HF.

In previous EF prediction research, many studies used ECG features and physiological parameters to predict EF^13^. However, by restricting the ECG features in the method, researchers may have ignored other important features. A previous study applied AI for predicting EF using the entire 12-lead ECG signal as a matrix and feeding into 2D-CNN for EF prediction^14^. But the crucial ECG leads were not identified.

In many ECG AI studies, researchers used 1D-CNN to classify ECG signals, such as for atrial fibrillation classification^4,15^. We compare 1D and 2D-CNNs in this study by establishing three simple 1D-CNN models and applied the 1D-CNN structure of the two papers on our dataset^16,17^ (Tables S4–S7). According to the above comparison, our 2D-CNN shows the highest accuracy in predicting systolic heart failure compared to the other three 1D-CNN models. Although such a result may be limited by the amount of training data, it still reveals that the 2D-CNN model may perform better than the 1D-CNN models when classifying using a single ECG compound.

Another study applied ECG CWT in a 2D-CNN for atrial arrhythmia detection but also focused on only one lead feature^18^. These studies have revealed that ECG CWT is a powerful tool for identifying abnormal ECG features. In our research, all ECG leads were used to expand all ECG features to the 2D-CNN to enhance the systolic HF classification capability. A key lead, useful for pre-screening patients for systolic HF, was identified by separately training the 12-leads in identical 2D-CNN models. The key lead cannot be identified without training the 12-leads concurrently.

Several ECG features have been used to assess the LV function, and the presence of prolonged QRS duration is a strong marker for diminished LV systolic function^19^. Our study also supported these findings. Under our algorithm, the widening of QRS and lower QRS amplitude imply the high probability of poor LVEF. As shown in supplemental Fig. 1, we also found other specific features that suggested poor contractility, including p wave amplitude, T amplitude and ST interval, which were also the possible indicators for poor LV contractility^20–22^. Our AI-assisted algorithm could combine those features and assess the possibility of LVEF of < 50% with good accuracy. On the other hand, our algorithm aimed to screen patients with reduced LVEF (LVEF < 50%) by using only 12-lead ECG, which is low-cost and easily-feasible. In many rural areas and developing countries, the difficult access to cardiologic care and imaging could cause under-diagnosis and treatment for heart failure. Our algorithm by converting 12-lead ECG to 2D images rather than raw data provides a portable, inexpensive test for ventricular systolic dysfunction. The early diagnosis of left ventricular dysfunction could permit early institution of effective therapies, such as beta-blockers, angiotensin receptor antagonists and implantable devices. Along with the smartphone-enable electrodes, the single-lead ECG could be acquired by using mobile applications. Our algorithm could also be incorporated into those applications to assess the ventricular function.

### Study limitation

The limitations of this research are deficiencies for patients with systolic HF and noise in all ECG data. For future studies to further improve screening accuracy, it is imperative to collect training data from hospitals to enhance the dataset continually. The new data can be used to improve our model’s performance. Furthermore, ECG signals should be kept clear and noise-free to prevent ECG slicing issue. Also, in this study, the prevalence of disease in our study cohort does not reflect prevalence in the general population. Thus, further research is needed to assess the utility of the given cutoffs in a general, ostensibly healthy population. At last, our dataset excluded patients with heart failure symptoms but with normal left ventricular systolic function. Therefore, heart failure patients with preserved LVEF may not be identified by using our algorithm.

## Conclusion

In this research, we revealed that ECG CWT spectra can expand all ECG features for 2D-CNN classification. With the comprehensive 12-lead ECG scoring method, systolic HF screening obtained an accuracy of over 0.94 in Lead V5 and V6 and an AUC of 0.98 in Lead I and V6. In addition, we found that the V6 lead is vital for detecting systolic HF. Overall, this study provided an effective and accurate screening method for predicting cardiac contractile dysfunction using 12-lead ECG images.

## Supplementary Information

Supplementary Information
