# Supplementary material for: Electrocardiogram lead selection for intelligent screening of patients with systolic heart failure
Source: Sci Rep. 2021 Jan 21;11:1948. doi: 10.1038/s41598-021-81374-6 (PMC7820471; doi:10.1038/s41598-021-81374-6)

**Electrocardiogram Lead Selection for Intelligent Screening of Patients with Systolic Heart Failure**

Yu-An Chiou^a^, Jhen-Yang Syu ^b^, Sz-Ying Wu^b^, Lian- Yu Lin^d^, Li Tzu Yi^e^, Ting-Tse Lin^b,c,d*^, Shien-Fong Lin^a,b*^

1. Department of Electrical and Computer Engineering, College of Electrical and Computer Engineering, National Chiao-Tung University, Hsinchu, Taiwan
2. Institute of Biomedical Engineering, College of Electrical and Computer Engineering, National Chiao-Tung University, Hsinchu, Taiwan
3. Department of Internal Medicine, National Taiwan University Hospital Hsin-Chu Branch, Hsin-Chu, Taiwan
4. Department of Internal Medicine, College of Medicine, National Taiwan University, Taipei, Taiwan
5. Department of Nursing, National Taiwan University Hospital Hsinchu Branch, Hsin-Chu, Taiwan

# Supplementary materials

**Table S1** CNN structure details. The full code can be found on GitHub. <https://github.com/yapuppy/ECG/settings>

| Layer (type) | Output Shape | Parameters |
| --- | --- | --- |
| Conv2d_1 (Conv2D) | (None, 200, 200, 32) | 416 |
| Activation_1 (Activation) | (None, 200, 200, 32) | 0 |
| Batch_normalization_1 | (None, 200, 200, 32) | 128 |
| Conv2d_2 (Conv2D) | (None, 200, 200, 32) | 9248 |
| Activation_2 (Activation) | (None, 200, 200, 32) | 0 |
| Batch_normalization_2 | (None, 200, 200, 32) | 128 |
| Max_pooling2d_1 | (None, 100, 100, 32) | 0 |
| Dropout_1 | (None, 100, 100, 32) | 0 |
| Conv2d_3 (Conv2D) | (None, 50, 50, 64) | 18496 |
| Activation_3 (Activation) | (None, 50, 50, 64) | 0 |
| Batch_normalization_3 | (None, 50, 50, 64) | 256 |
| Conv2d_4 (Conv2D) | (None, 50, 50, 64) | 36928 |
| Activation_4 (Activation) | (None, 50, 50, 64) | 0 |
| Batch_normalization_4 | (None, 50, 50, 64) | 256 |
| Max_pooling2d_2 | (None, 25, 25, 64) | 0 |
| Dropout_2 (Dropout) | (None, 25, 25, 64) | 0 |
| Conv2d_5 (Conv2D) | (None, 13, 13, 128) | 73856 |
| Activation_5 (Activation) | (None, 13, 13, 128) | 0 |
| Batch_normalization_5 | (None, 13, 13, 128) | 512 |
| Conv2d_6 (Conv2D) | (None, 13, 13, 128) | 147584 |
| Activation_6 (Activation) | (None, 13, 13, 128) | 0 |
| Batch_normalization_6 | (None, 13, 13, 128) | 512 |
| Max_pooling2d_3 | (None, 6, 6, 128) | 0 |
| Dropout_3 (Dropout) | (None, 6, 6, 128) | 0 |
| Flatten_1 (Flatten) | (None, 4608) | 0 |
| Dense_1 (Dense) | (None, 2) | 9218 |
| Total params: 297,538  Trainable params: 296,642  Non-trainable params: 896 | | |

**Table S2.** 2D-CNN hyperpatameters list.

| Hyper-parameter | Value |
| --- | --- |
| Layer type | 2D convolutional layers |
| Learning rate | 0.0004 |
| Number of epochs | 500 |
| Early stop patience | 50 |
| Batch size | 20 |
| Loss function | Crossentropy |
| Optimizer | Adam |

**Table S3.** The 12-lead ECG 2D-CNN cross-validation result can train 1,260 samples and validate 140 samples.

| Cross validation | Lead I | Lead II | Lead III | aVF | aVR | aVL | V1 | V2 | V3 | V4 | V5 | V6 |
| --- | --- | --- | --- | --- | --- | --- | --- | --- | --- | --- | --- | --- |
| Dataset 1 | 72.34% | 66.67% | 71.88% | 76.42% | 83.33% | 79.10% | 75.41% | 73.77% | 82.05% | 75.35% | 78.99% | 96.06% |
| Dataset 2 | 74.47% | 74.07% | 76.56% | 68.29% | 81.16% | 84.33% | 73.55% | 77.87% | 76.92% | 74.65% | 86.96% | 88.19% |
| Dataset 3 | 70.92% | 71.85% | 69.29% | 75.41% | 80.43% | 76.12% | 78.51% | 74.59% | 85.47% | 74.65% | 84.06% | 82.68% |
| Dataset 4 | 78.01% | 62.96% | 63.78% | 72.13% | 83.33% | 83.46% | 74.38% | 79.51% | 78.63% | 77.46% | 78.83% | 90.48% |
| Dataset 5 | 73.76% | 71.85% | 62.99% | 74.59% | 78.99% | 78.20% | 71.07% | 73.77% | 78.63% | 82.39% | 76.64% | 86.51% |
| Dataset 6 | 77.86% | 65.93% | 66.14% | 71.31% | 86.13% | 80.45% | 71.90% | 85.12% | 79.49% | 82.39% | 77.37% | 88.10% |
| Dataset 7 | 76.43% | 69.63% | 66.14% | 67.21% | 83.94% | 72.18% | 84.30% | 76.86% | 78.63% | 78.87% | 81.02% | 90.48% |
| Dataset 8 | 81.43% | 66.67% | 68.50% | 83.61% | 79.56% | 78.95% | 80.17% | 83.47% | 82.91% | 84.51% | 79.56% | 86.51% |
| Dataset 9 | 83.57% | 66.42% | 76.38% | 72.13% | 76.64% | 78.95% | 71.90% | 76.03% | 81.03% | 80.99% | 89.05% | 90.48% |
| Dataset 10 | 73.57% | 71.64% | 67.72% | 72.13% | 81.02% | 80.45% | 80.99% | 75.21% | 78.45% | 80.99% | 81.02% | 91.27% |
| average(+/- std) | 76.24% (+/- 3.83%) | 68.77% (+/- 3.35%) | 68.94% (+/- 4.49%) | 73.32% (+/- 4.38%) | 81.45% (+/- 2.63%) | 79.22% (+/- 3.28%) | 76.22% (+/- 4.29%) | 77.62% (+/- 3.77%) | 80.22% (+/- 2.47%) | 79.23% (+/- 3.38%) | 81.35% (+/- 3.89%) | 89.07% (+/- 3.40%) |

**Table S4.** Our 1D-CNN structure details.

| Layer (type) | Output Shape | Parameters |
| --- | --- | --- |
| Input layer | (None, 1000, 2) | 396 |
| Conv1d_1 (Conv1D) | (None, 993, 100) | 1700 |
| Conv1d_1 (Conv1D) | (None, 986, 100) | 80100 |
| Max_pooling2d_1 | (None, 328, 100) | 0 |
| Dropout_1 | (None, 328, 100) | 0 |
| Conv1d_1 (Conv1D) | (None, 321, 200) | 160200 |
| Conv1d_1 (Conv1D) | (None, 314, 200) | 320200 |
| Max_pooling2d_1 | (None, 200) | 0 |
| Dropout_1 | (None, 200) | 0 |
| Dense layer | (None, 2) | 402 |

**Table S5.** Our 1D-CNN hyperparameters.

| Hyper-parameter | Value |
| --- | --- |
| Layer type | 1D convolutional layers |
| Learning rate | 0.0004 |
| Number of epochs | 500 |
| Early stop patience | 50 |
| Batch size | 200 |
| Loss function | Crossentropy |
| Optimizer | Adam |

| **Cross validation** | **Lead I** | **Lead II** | **Lead III** | **aVF** | **aVR** | **aVL** | **V1** | **V2** | **V3** | **V4** | **V5** | **V6** |
| --- | --- | --- | --- | --- | --- | --- | --- | --- | --- | --- | --- | --- |
| **Dataset 1** | 54.35% | 55.98% | 56.52% | 55.43% | 55.43% | 56.52% | 66.30% | 75.54% | 72.28% | 71.20% | 60.87% | 59.24% |
| **Dataset 2** | 54.35% | 52.72% | 54.89% | 54.35% | 54.35% | 54.35% | 65.76% | 74.46% | 71.74% | 64.67% | 63.04% | 59.24% |
| **Dataset 3** | 55.98% | 55.43% | 54.89% | 54.35% | 54.89% | 54.35% | 69.57% | 76.63% | 72.28% | 70.65% | 66.30% | 61.41% |
| **Dataset 4** | 53.26% | 54.35% | 53.80% | 54.35% | 55.98% | 55.43% | 63.04% | 73.37% | 71.20% | 71.74% | 59.24% | 60.33% |
| **Dataset 5** | 54.35% | 57.61% | 59.78% | 55.43% | 55.43% | 56.52% | 67.93% | 73.91% | 76.09% | 72.28% | 61.41% | 57.07% |
| **Dataset 6** | 55.43% | 52.72% | 57.61% | 54.89% | 58.15% | 55.43% | 71.20% | 73.91% | 72.28% | 70.65% | 61.96% | 56.52% |
| **Dataset 7** | 55.43% | 57.07% | 56.52% | 55.43% | 54.89% | 57.07% | 70.65% | 72.28% | 70.65% | 71.20% | 62.50% | 61.41% |
| **Dataset 8** | 54.35% | 57.07% | 58.15% | 56.52% | 55.98% | 55.98% | 72.83% | 78.80% | 76.63% | 67.93% | 60.87% | 60.87% |
| **Dataset 9** | 54.35% | 54.35% | 55.43% | 54.35% | 53.80% | 54.89% | 68.48% | 75.00% | 73.37% | 67.39% | 63.04% | 61.96% |
| **Dataset 10** | 53.80% | 55.98% | 58.70% | 55.43% | 56.52% | 57.07% | 66.30% | 73.37% | 71.20% | 67.39% | 61.41% | 58.15% |
| **average(+/- std)** | 54.57% (+/- 0.78%) | 55.33% (+/- 1.66%) | 56.63% (+/- 1.82%) | 55.05% (+/- 0.69%) | 55.54% (+/- 1.16%) | 55.76% (+/- 0.98%) | 68.21% (+/- 2.80%) | 74.73% (+/- 1.79%) | 72.77% (+/- 1.94%) | 69.51% (+/- 2.36%) | 62.07% (+/- 1.78%) | 59.62% (+/- 1.80%) |

**Table S6.** Our 1D-CNN 10 cross-validation results.

**Table S7**. Two 1D-CNN structure compare to our 1D-CNN and 2D-CNN model.

| 10 fold-Cross validation-  average(+/- std) | Lead I | Lead II | Lead III | aVF | aVR | aVL | V1 | V2 | V3 | V4 | V5 | V6 |
| --- | --- | --- | --- | --- | --- | --- | --- | --- | --- | --- | --- | --- |
| 1D-CNN  (Kiranyaz et al. 2016) | 54.24% (+/- 0.22%) | 54.24% (+/- 0.22%) | 54.24% (+/- 0.22%) | 54.24% (+/- 0.22%) | 54.24% (+/- 0.22%) | 54.24% (+/- 0.22%) | 54.24% (+/- 0.22%) | 54.24% (+/- 0.22%) | 54.24% (+/- 0.22%) | 54.24% (+/- 0.22%) | 54.24% (+/- 0.22%) | 54.24% (+/- 0.22%) |
| 1D-CNN  (Hsieh et al. 2020) | 54.35% (+/- 0.34%) | 55.38% (+/- 1.15%) | 57.61% (+/- 1.90%) | 54.51% (+/- 0.25%) | 55.54% (+/- 1.61%) | 56.20% (+/- 1.06%) | 67.93% (+/- 2.00%) | 74.57% (+/- 2.73%) | 73.15% (+/- 1.95%) | 67.88% (+/- 2.71%) | 61.85% (+/- 1.68%) | 60.65% (+/- 3.02%) |
| 1D-CNN  (our model) | 54.57% (+/- 0.78%) | 55.33% (+/- 1.66%) | 56.63% (+/- 1.82%) | 55.05% (+/- 0.69%) | 55.54% (+/- 1.16%) | 55.76% (+/- 0.98%) | 68.21% (+/- 2.80%) | 74.73% (+/- 1.79%) | 72.77% (+/- 1.94%) | 69.51% (+/- 2.36%) | 62.07% (+/- 1.78%) | 59.62% (+/- 1.80%) |
| 2D-CNN  (our model) | 76.24% (+/- 3.83%) | 68.77% (+/- 3.35%) | 68.94% (+/- 4.49%) | 73.32% (+/- 4.38%) | 81.45% (+/- 2.63%) | 79.22% (+/- 3.28%) | 76.22% (+/- 4.29%) | 77.62% (+/- 3.77%) | 80.22% (+/- 2.47%) | 79.23% (+/- 3.38%) | 81.35% (+/- 3.89%) | 89.07% (+/- 3.40%) |

**Figure S1.** There are specific features suggested poor contractility, including p wave amplitude, T amplitude, ST interval and QRS duration, which were also the possible indicators for poor LV contractility.


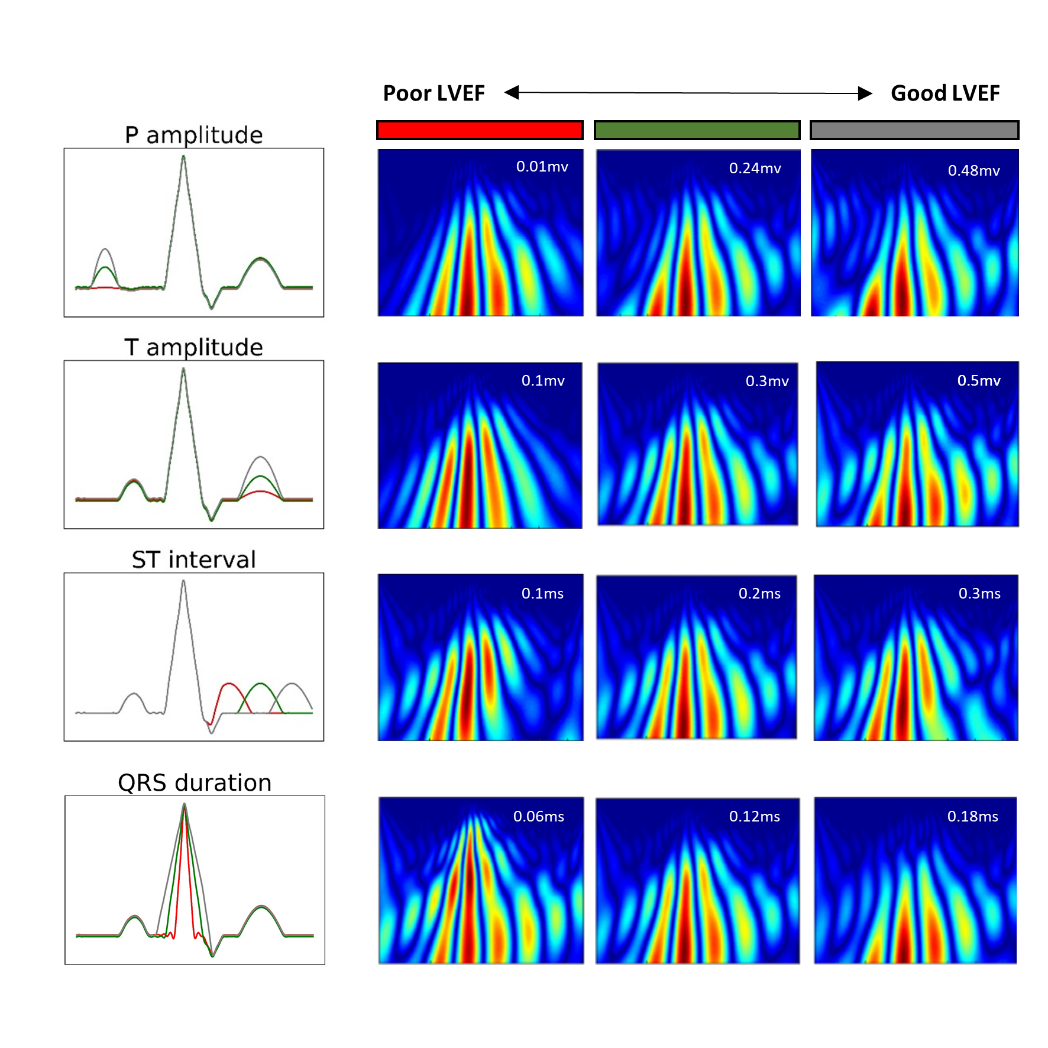

Supplement: Supplementary file 1 — Supplementary Information [file 41598_2021_81374_MOESM1_ESM.docx]
